# External Validation of Clinical Risk Scores and Machine Learning Models for Predicting 30-Day Cardiovascular Risk After Noncardiac Surgery: The PERICARE Study

**DOI:** 10.3390/jcm15176848

**Published:** 2026-09-04

**Authors:** Aslan Erdoğan, Şeyma Yeşil, Gamze Gençol Akçay, Ufuk Sali Halil, İhsan Demirtaş, Ezgi Alp, Berat Erdem, Fatma Ekici, Tuğba Öztürk, İpek Göker, Mustafacan Kılıçoğlu, Hüseyin Akgün, Yusuf İnci, Utku Uluköksal, Furkan Fatih Yücedağ, Alihan Ayata, Baran Yılmaz, Taylan Akgün, Selim Topçu, Can Yücel Karabay

**Affiliations:** 1 Department of Cardiology, Cam and Sakura Training and Research Hospital, 34480 Istanbul, Turkeyhuseyin.akgun1@saglik.gov.tr (H.A.);; 2 Department of Cardiology, University of Health Sciences, Dr. Siyami Ersek Thoracic and Cardiovascular Surgery Training and Research Hospital, 34668 Istanbul, Turkey; 3 Department of Cardiology, Medical Faculty, Yeditepe University, 34755 Istanbul, Turkey; 4 Department of Cardiology, Liv Hospital Gaziantep, 27080 Gaziantep, Turkey

**Keywords:** artificial intelligence, clinical prediction model, deep learning, perioperative cardiovascular risk, noncardiac surgery

## Abstract

**Background**: Machine learning (ML) has emerged as a promising approach for preoperative cardiovascular risk prediction; however, the generalizability of ML models across institutions remains uncertain. Moreover, comprehensive head-to-head comparisons between ML algorithms and established clinical risk scores for predicting 30-day major adverse cardiac events (MACE) are scarce. We therefore evaluated the performance and external transportability of multiple ML models across independent centers and compared their predictive accuracy with validated benchmark clinical risk scores. **Methods**: In a site-separated two-center cohort (derivation *n =* 707, 27 MACE; external validation *n =* 378, 38 MACE), ten algorithms trained on preoperative variables were externally validated without refitting and benchmarked against the American university of Beirut-HAS2 (AUB-HAS2), American society of anesthesiologists (ASA), and revised cardiac risk index (RCRI). We assessed AUROC, calibration, Brier score, and decision-curve net benefit, with paired bootstrap comparisons, DeLong testing, IDI, and NRI. **Results**: Among the ML models, no single algorithm consistently outperformed the others across all performance metrics. Naive Bayes achieved the highest external discrimination (AUROC 0.738, 95% CI 0.668–0.804) but showed poor calibration and threshold-dependent clinical utility. Gradient Boosting showed the most favorable balance of discrimination and calibration slope (AUROC 0.707; calibration slope 0.991), although absolute risk remained underestimated in external validation, whereas HistGradient Boosting yielded the best overall probability prediction, with the lowest Brier score (0.087) and the greatest decision-curve net benefit at clinically relevant risk thresholds. However, in external validation, no ML model demonstrated statistically significant AUROC superiority over the AUB-HAS2 score (all *p* > 0.05), although several models modestly outperformed the RCRI. **Conclusions**: In this site-separated external validation, ML models showed metric-dependent performance but no discrimination advantage over the AUB-HAS2 index. Given low event counts, flexible-model results are hypothesis-generating. These findings provide a cautionary, reproducible benchmark; local recalibration and prospective evaluation are prerequisites before clinical deployment.

## 1. Introduction

Cardiovascular complications after noncardiac surgery remain a major cause of perioperative morbidity, mortality, prolonged hospitalization, and healthcare resource utilization. Accordingly, contemporary North American and European guidelines recommend structured preoperative cardiovascular risk assessment incorporating patient comorbidities, surgical risk, functional capacity, and the selective use of biomarkers or imaging when these findings are expected to influence perioperative management [1,2]. Despite these recommendations, accurately estimating an individual patient’s cardiovascular risk remains challenging. Risk assessment tools must be sufficiently simple for routine clinical use while maintaining adequate predictive performance across increasingly older, frailer, and multimorbid surgical populations with diverse case mixes and healthcare settings.

Several clinical prediction models have therefore been developed to support perioperative risk stratification. The Revised Cardiac Risk Index (RCRI) remains the most widely adopted model because of its simplicity, transparency, and ease of implementation [3]. The American Society of Anesthesiologists (ASA) Physical Status Classification is also routinely incorporated into perioperative assessment, although it was not originally designed as a cardiovascular risk model and is limited by interobserver variability [4]. More recently, the American University of Beirut-HAS2 (AUB-HAS2) cardiovascular risk index has demonstrated favorable discrimination across a broad range of surgical populations [5,6]. Although these scores provide practical and clinically validated benchmarks, their performance may vary across institutions owing to differences in patient characteristics, emergency surgical burden, comorbidity profiles, and local clinical practice. Consequently, independent external validation remains essential before widespread implementation in new healthcare settings.

Machine learning (ML) has been proposed as a complementary approach to conventional clinical prediction because flexible algorithms can represent nonlinear associations and higher-order interactions among routinely collected preoperative variables. Recent scientific guidance from the American Heart Association has recognized the potential of artificial intelligence for cardiovascular risk assessment while emphasizing that rigorous external validation, calibration assessment, generalizability, and prospective clinical evaluation remain prerequisites for routine implementation [7], and systematic comparative evidence has not demonstrated consistent superiority of ML over well-specified regression models for structured clinical prediction [8]. However, perioperative prediction represents a particularly challenging environment for ML. Event rates are relatively low, missing data are often informative, calibration frequently deteriorates across institutions, and improvements in discrimination do not necessarily translate into clinically useful or transportable predictions. Accordingly, contemporary methodological guidance recommends that ML models be evaluated not only by discrimination but also by calibration, overall prediction error, clinical utility, and, most importantly, rigorous external validation [9,10]. For context, large observational studies have demonstrated the feasibility of data-driven perioperative risk prediction at scale: a nationwide analysis of 133,634 gastrointestinal operations evaluated ML-based prediction and stratification of perioperative cardiovascular events [11], and a recent multicenter MySurgeryRisk analysis of 508,097 surgical encounters from 366,875 patients across 14 institutions evaluated automated prediction of major postoperative complications and in-hospital mortality [12]. These studies differ from the present work in target outcomes, predictor scope, populations, and validation frameworks, and large sample size does not by itself establish transportability across institutions. The PERICARE study therefore addresses a complementary question: whether models developed at one institution retain discrimination, calibration, and clinical utility in an independent institution, and whether they provide meaningful incremental value over readily available clinical scores.

Against this background, we conducted the PERICARE study to develop and externally validate ten machine-learning algorithms for predicting 30-day major adverse cardiovascular events after noncardiac surgery using a deliberately site-separated two-center design. We benchmarked each model against established clinical risk scores, including RCRI, ASA Physical Status, and AUB-HAS2, to determine whether ML provides meaningful incremental value over readily available clinical tools in routine perioperative practice. To maximize methodological rigor and clinical relevance, validation was performed in an entirely independent institution rather than by random data splitting, reporting followed the TRIPOD+AI statement, and risk of bias and applicability were evaluated using PROBAST+AI [13,14]. PERICARE is a retrospective, consecutive, site-separated two-center cohort of adults referred for preoperative cardiovascular assessment before noncardiac surgery between February 2023 and May 2025; Cam and Sakura Training and Research Hospital served as the derivation site, and Dr Siyami Ersek Thoracic and Cardiovascular Surgery Training and Research Hospital served as the independent external validation site. The study was designed primarily to evaluate cross-institutional transportability rather than performance after random within-cohort splitting.

## 2. Methods

### 2.1. Study Design, Population, and Endpoints

This retrospective observational analysis included 1085 consecutive patients referred to our centers for preoperative assessment before noncardiac surgery between February 2023 and May 2025. Patients were excluded if they met at least one of the following criteria: cardiac surgery, pregnancy, or missing/insufficient data. Follow-up data were obtained from the national health registration system or by telephone interview. This study was conducted in accordance with the Declaration of Helsinki. The ethics committee of our hospital approved the study protocol (date: 25 May 2025, decision number: 186). The need for written informed consent was waived because of the retrospective study design.

**Figure 1:**
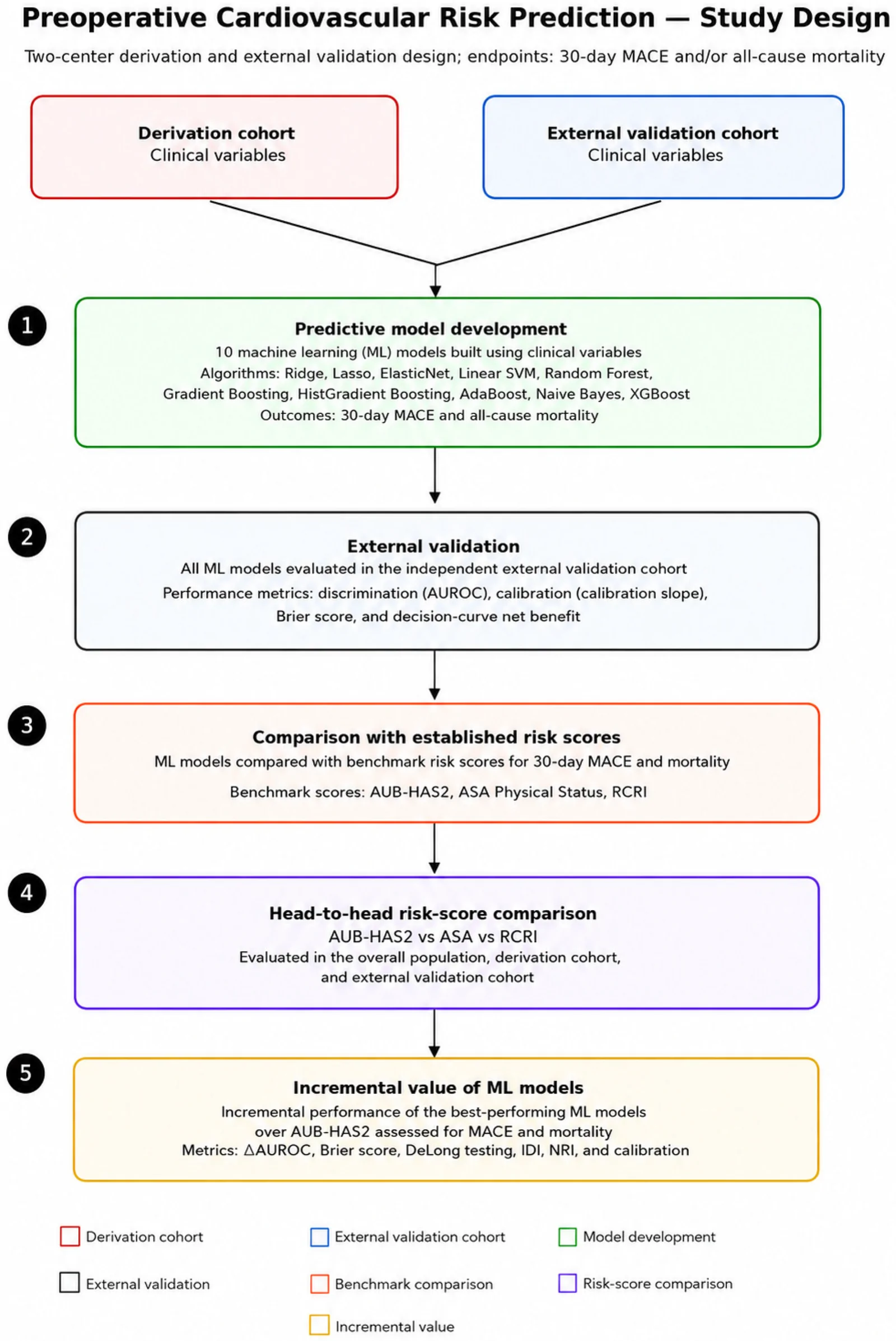
Study design and analysis framework. The site-separated derivation and external validation cohorts were used for clinical ML model development and validation, benchmark risk-score comparison.

This was a two-center observational model-development and external validation study of adults evaluated before noncardiac surgery. Cam-Sakura Hospital served as the derivation cohort and Siyami Ersek Hospital served as the external validation cohort. This separation was chosen to provide a clinically meaningful test of transportability across institutions, patient mix, and practice patterns rather than a random split that would share site-level artifacts across training and testing. The overall study workflow is shown in Figure 1.

We followed the reporting and validation principles emphasized in contemporary prediction-model guidance, including explicit separation of development and validation data, assessment of discrimination and calibration, and transparent reporting of model comparisons and intended use [13,14].

**Endpoints and outcome definitions**

The primary endpoint was 30-day MACE, defined a priori as a composite of all-cause death, non-fatal cardiac arrest, myocardial infarction or acute coronary syndrome, stroke, and new or worsening heart failure occurring within 30 days after surgery. All-cause mortality at 30 days was analyzed as a separate endpoint. Component definitions followed the 2022 ESC Guidelines on non-cardiac surgery and the relevant contemporary consensus standards, as detailed below [2].

Death: All-cause death was defined as death from any cause within 30 days of surgery.

Non-fatal cardiac arrest: Cardiac arrest was defined, per the American Heart Association/American College of Cardiology consensus [15], as the sudden cessation of cardiac activity with unresponsiveness, absent or agonal breathing, and no signs of circulation. A non-fatal (resuscitated) cardiac arrest required successful resuscitation with return of spontaneous circulation, evidenced by restoration of organized electrical and mechanical activity producing a measurable pulse and blood pressure; fatal arrests were classified under death.

Myocardial infarction and acute coronary syndrome: Myocardial infarction was defined according to the Fourth Universal Definition of Myocardial Infarction, requiring a rise and/or fall of cardiac troponin with at least one value above the 99th-percentile upper reference limit together with evidence of acute myocardial ischemia (ischemic symptoms, new ischemic electrocardiographic changes, development of pathological Q waves, imaging evidence of new loss of viable myocardium or a new regional wall-motion abnormality, or identification of intracoronary thrombus). Acute coronary syndrome encompassed unstable angina and non–ST-segment and ST-segment elevation acute coronary syndromes [16].

Stroke: Stroke was defined according to the American Heart Association/American Stroke Association tissue-based definition as an acute episode of focal neurological dysfunction caused by central nervous system infarction or hemorrhage, confirmed either by objective imaging or pathological evidence of injury within a vascular territory, or by clinical symptoms of focal ischemia persisting for 24 h or longer or resulting in death, after exclusion of non-vascular causes. Events were classified as ischemic, hemorrhagic, or of undetermined type [17].

Heart failure: New or worsening heart failure was defined as new-onset or decompensated signs and symptoms of heart failure requiring initiation or intensification of treatment (for example, intravenous diuretics, vasodilators, or inotropes) or unplanned hospitalization or prolongation of stay for heart failure, supported where available by objective evidence of congestion on examination, imaging, or natriuretic peptide concentrations.

### 2.2. Clinical Predictors and Benchmark Comparators

Candidate predictors were selected from variables routinely available before surgery: age, sex, hemoglobin, creatinine, renal function surrogates, left ventricular ejection fraction, diabetes, insulin-treated diabetes, hypertension, coronary artery disease, prior myocardial infarction, preoperative heart failure history, chronic kidney disease, cerebrovascular disease, peripheral arterial disease, atrial fibrillation, functional capacity, surgical risk category, emergency status, and surgery type. Troponin and natriuretic peptide values were summarized but were not included as primary predictors because missingness was extensive.

Benchmark risk scores (AUB-HAS2, ASA physical status, and RCRI) were calculated as available in the dataset. They were evaluated as comparator models overall and separately in the derivation and external validation cohorts using the same endpoint definition as the ML models. Because Brier scores, calibration metrics, and decision-curve analyses require predicted probabilities rather than ordinal score points, each score was converted to a probability using a one-variable logistic model (30-day MACE regressed on the score as a linear term) fitted in the derivation cohort only; this fixed mapping was then applied unchanged to the external validation cohort, without recalibration of any kind in the external data. Discrimination metrics were computed from the score rankings and are unaffected by this monotonic transformation.

### 2.3. Clinical Machine-Learning Model Development

Ten clinical ML algorithms were developed in the derivation cohort and externally validated without refitting: ridge logistic regression, lasso logistic regression, elastic-net logistic regression, linear support-vector machine, random forest, gradient boosting, histogram gradient boosting, AdaBoost, naïve Bayes, and XGBoost. No single ML algorithm was prespecified as the primary model. All models were assessed across the same external validation framework, and interpretation was based on the full performance profile rather than AUROC alone.

The modeling pipeline used training-only preprocessing to avoid information leakage. No patient was excluded for missing predictor values. Continuous predictors were imputed with the development-cohort median and accompanied by binary missingness indicators; binary predictors were imputed with the development-cohort mode with missingness indicators; and categorical predictors were imputed with the most frequent development-cohort category and one-hot encoded, with categories unseen during development mapped to zero at validation. Continuous predictors were standardized using development-cohort parameters for the penalized logistic models, the linear support-vector machine, and naive Bayes, whereas tree-based ensembles used unscaled features. All imputation, scaling, and encoding parameters were learned exclusively in the development cohort and applied unchanged to the external validation cohort. Given the low number of development events, extensive data-driven hyperparameter searching was deliberately avoided. Only the ridge regularization strength was tuned, once, within the development cohort, using a stratified 5-fold cross-validated grid search over a fixed logarithmic grid (10^−3^ to 10^2^) with log-loss as the selection criterion (selected C = 0.72); the remaining nine algorithms used fixed, prespecified, conservative hyperparameters with no data-driven search (Supplementary Table S8). Class imbalance was addressed with balanced class weights where supported, and the probability outputs of each algorithm were calibrated with a Platt (sigmoid) layer fitted by internal 5-fold splitting within the development cohort. Nested cross-validation was not performed: because hyperparameters were fixed a priori for nine of the ten algorithms and tuned once for ridge, tuning-related optimism could affect only the development-stage internal estimates, not the external validation results, which were obtained in a cohort never used for any tuning, selection, calibration, or threshold decision. Development-stage performance was estimated using repeated stratified 5-fold cross-validation (10 repeats), with out-of-fold predictions averaged across repeats. The predictor specification was finalized within the development cohort: after an initial broader specification based on the full candidate list produced unstable coefficient estimates under internal cross-validation, a parsimonious specification of nine routinely available preoperative variables (age, sex, hemoglobin, creatinine, functional capacity, diabetes mellitus, prior myocardial infarction, preoperative heart failure history, and high-risk surgical category; 15 design features after encoding and missingness indicators) was adopted before any external testing. Final fitted models were applied once to the locked external validation cohort without refitting or recalibration, and a fixed random seed was used throughout. Because event prevalence was low, probability outputs were interpreted through calibration metrics and decision-curve analyses rather than through class labels.

The algorithm set included both transparent shrinkage models and more flexible nonlinear models. Penalized logistic models provide shrinkage and interpretability under low event counts, whereas tree-based ensembles can capture nonlinearities and interactions but may be less stable and more difficult to calibrate. This design is consistent with prior evidence that complex ML does not necessarily outperform well-specified regression models in structured clinical prediction tasks [8,18,19,20,21,22].

### 2.4. Statistical Analysis

Continuous variables are summarized as median and interquartile range; categorical variables are summarized as counts and percentages. Baseline characteristics were not subjected to hypothesis testing because the purpose was descriptive characterization of derivation, validation, and event strata rather than causal inference.

Discrimination was quantified by AUROC with bootstrap 95% confidence intervals. Calibration was assessed using calibration slope, calibration intercept, observed-to-expected ratio, and calibration curves. Overall accuracy of probabilistic prediction was assessed using Brier score and log loss. Decision-curve analysis estimated net benefit across clinically relevant threshold probabilities and at prespecified thresholds of 5%, 10%, and 15%, reflecting the range where perioperative escalation, monitoring, or additional testing might be considered [9,10,23,24,25]. These threshold probabilities were selected as clinically interpretable reference points spanning the low-to-moderate risk range in which intensified perioperative monitoring or additional evaluation might be considered in this referred population; at a 5% threshold the implied exchange rate is approximately one event identified per 19 patients flagged, with 10% and 15% representing progressively more selective escalation. They served as illustrative anchors rather than guideline-mandated treatment cutoffs, and decision curves were interpreted across the full threshold range, including treat-all and treat-none strategies.

For head-to-head model comparisons in the external validation cohort, paired bootstrap resampling was used to preserve within-patient correlation among predicted probabilities. All clinical ML models were compared with benchmark scores, including AUB-HAS2, ASA, and RCRI. AUROC differences were assessed using DeLong testing, and incremental discrimination and reclassification were summarized using integrated discrimination improvement (IDI) and net reclassification improvement (NRI) [10,25,26]. Because biomarker-based postoperative myocardial injury is strongly associated with mortality but was not uniformly available in this dataset, biomarker analyses were descriptive only [27,28].

Analyses were performed using Python (version 3.12.9; Python Software Foundation, Wilmington, DE, USA) utilizing the open-source libraries pandas (version 2.3.3), NumPy (version 2.4.2), scikit-learn (version 1.8.0), statsmodels (version 0.15.0), joblib (version 1.5.3), and matplotlib (version 3.11.0). The externally validated PERICARE-ML models were implemented as a research-only web calculator at https://www.pericaremace.com/ (accessed on 8 July 2026) This calculator is provided solely for research transparency and reproducibility; it has not been recalibrated or prospectively evaluated for any individual patient and is not intended for clinical decision-making.

## 3. Results

### 3.1. Study Population and Baseline Characteristics

The analytic clinical cohort included 1085 patients, with 707 in the derivation cohort and 378 in the external validation cohort. There were 65 total 30-day MACEs: 27 in the derivation cohort and 38 in the external validation cohort. Patients in the external validation cohort were older, had lower functional capacity, and had a higher proportion of emergency surgery. MACE-positive patients were older, more often had chronic kidney disease, cerebrovascular disease, atrial fibrillation, lower hemoglobin, lower functional capacity, and higher AUB-HAS2 scores (Table 1). Missingness was modest for core clinical variables but very high for troponin and NT_proBNP, supporting their exclusion from primary ML model development (Supplementary Table S1). The complete baseline distribution used for modeling interpretation is provided in Supplementary Table S2. With 27 derivation events across approximately 20 candidate predictors, the events-per-variable ratio was low. This was anticipated and addressed a priori by prioritising penalised and shrinkage-based models, by validating fitted models in an independent cohort without refitting, and by interpreting the full discrimination–calibration–net-benefit profile rather than AUROC alone; estimates from flexible nonlinear models should accordingly be read as hypothesis-generating and are reported with bootstrap confidence intervals to convey their uncertainty.

The study design and analysis hierarchy are depicted in Figure 1. Clinical ML models were developed in the derivation cohort and tested in the external validation cohort; benchmark scores were evaluated overall and by cohort; and mortality probabilities were added to clinical models for incremental-value testing.

### 3.2. Clinical Machine-Learning Models

**Figure 2:**
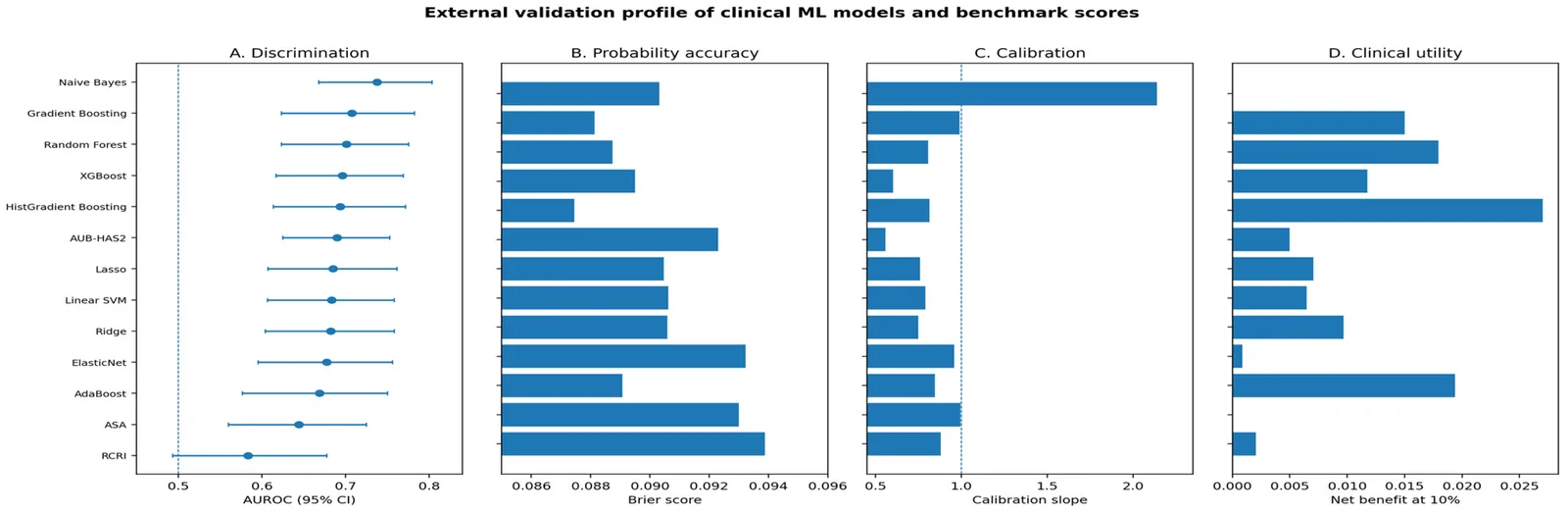
External validation profile of clinical ML models and benchmark scores. (**A**) Discrimination. Dots show the point estimate of the AUROC for each model/score; horizontal error bars represent the 95% confidence interval. The vertical dashed line at AUROC = 0.50 indicates the reference level for no discrimination. (**B**) Probability accuracy. Bars show the Brier score for each model/score; lower values indicate more accurate probability estimates. No lines or dots appear in this panel. (**C**) Calibration. Bars show the calibration slope for each model/score. The vertical dashed line at a slope of 1.0 represents perfect calibration (predicted probabilities matching observed outcomes on average); values below 1.0 indicate overfitting/overly extreme predictions, and values above 1.0 indicate underfitting/overly conservative predictions. (**D**) Clinical utility. Bars show the net benefit of each model/score at a 10% risk threshold, reflecting the trade-off between true- and false-positive classifications at that decision threshold.

No single ML model was prespecified as primary; therefore, external validation was interpreted across discrimination, Brier score, calibration, and decision-curve utility. Naive Bayes had the highest external AUROC (0.738; 95% CI: 0.668–0.804) and the highest net benefit at the 5% threshold (0.068), but its calibration slope was 2.139 and net benefit was zero at the 10% and 15% thresholds, indicating that ranking performance did not translate into reliable probability-based utility across thresholds (Table 2; Figure 2).

**Figure 3:**
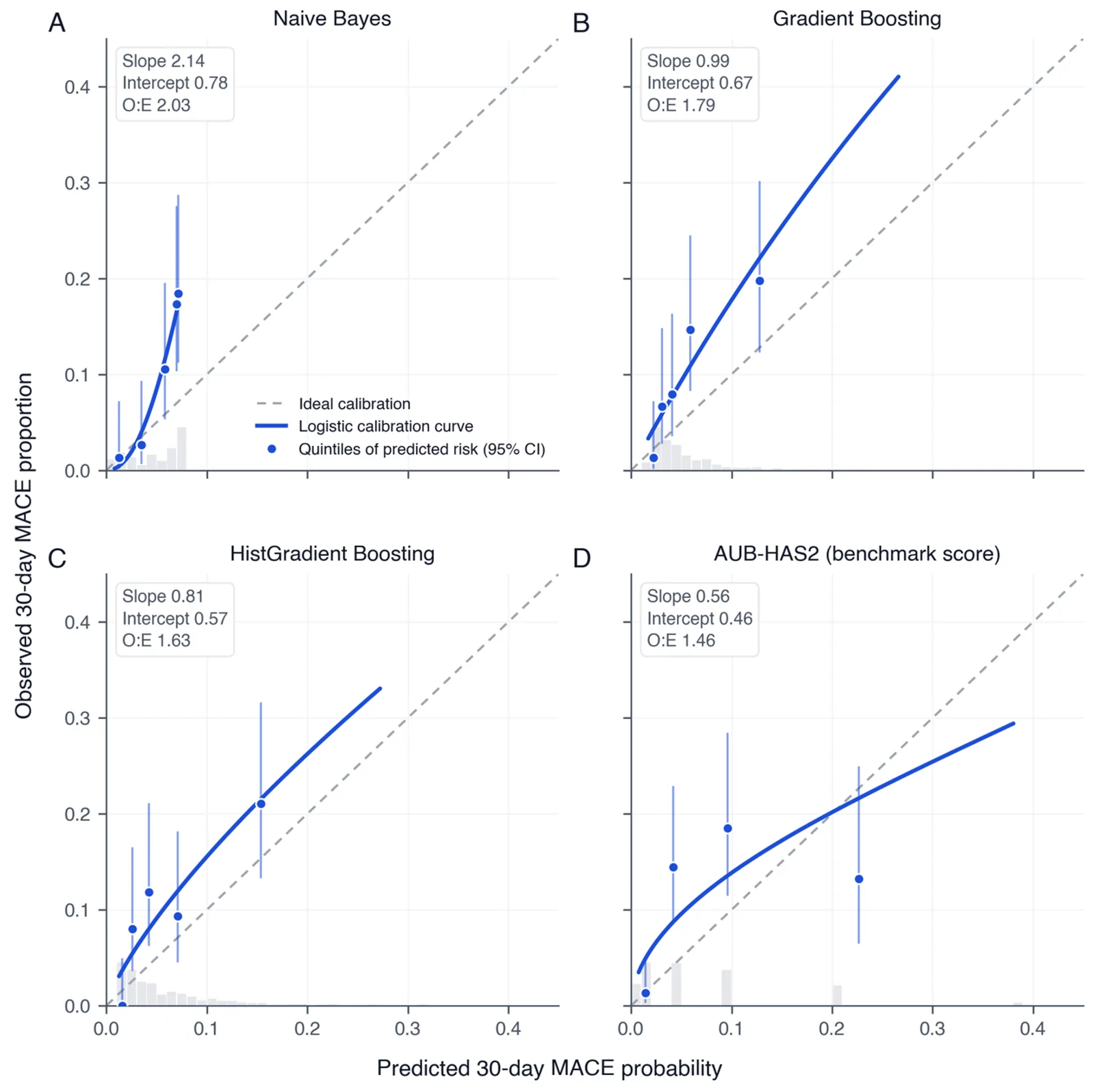
Panels show calibration for (**A**) Naive Bayes, (**B**) Gradient Boosting, (**C**) HistGradient Boosting, and (**D**) AUB-HAS2 (benchmark score). In each panel, the gray dashed diagonal line represents ideal calibration (predicted risk equal to observed risk). The solid blue line is the logistic (flexible) calibration curve, estimated by regressing observed 30-day MACE outcomes on predicted risk. Blue dots show the observed event proportion within each quintile of predicted risk, with vertical blue lines representing 95% confidence intervals. The gray histogram along the x-axis shows the distribution (density) of predicted risk values across the validation cohort. Inset text reports the calibration slope, calibration intercept, and observed-to-expected (O:E) event ratio for each model.

Gradient Boosting had the highest AUROC after Naive Bayes (0.707; 95% CI: 0.623–0.783) and a calibration slope close to 1.0 (0.991), with Brier score 0.088 and net benefit 0.015 and 0.011 at 10% and 15%. HistGradient Boosting had slightly lower AUROC (0.694; 95% CI: 0.613–0.772) but the lowest Brier score (0.087), the most favorable scaled Brier/IPA profile, and the highest net benefit at 10% (0.027) and 15% (0.011). Random Forest also performed well (AUROC 0.701; Brier 0.089; net benefit 0.050 at 5%). Ridge, Lasso, and Linear SVM were similar to each other, with AUROC 0.683 to 0.685 and Brier approximately 0.090 to 0.091 (Table 2; Supplementary Tables S4 and S5, and Supplementary Figure S4). External calibration curves for the three ML models with the most favorable metric-specific profiles (Naive Bayes, Gradient Boosting, and HistGradient Boosting) and for the AUB-HAS2 benchmark are shown in Figure 3. All four predictors underestimated absolute risk in the external cohort (observed-to-expected ratios 1.46 to 2.03), consistent with the higher event rate in the validation center. Gradient Boosting tracked the ideal line most closely (calibration slope 0.991), whereas Naive Bayes produced a severely compressed predicted-risk range, with no patient assigned an external predicted probability above 7.2%, explaining both its steep calibration slope (2.139) and its zero net benefit at the 10% and 15% thresholds.

In paired bootstrap comparisons, no ML model showed statistically significant AUROC superiority over the AUB-HAS2 benchmark. Gradient Boosting had ΔAUROC 0.017 versus AUB-HAS2 (*p* = 0.600), HistGradient Boosting had ΔAUROC 0.004 (*p* = 0.904), and Naive Bayes had ΔAUROC 0.048 (*p* = 0.235). Several models showed nominal discrimination improvements over RCRI, including Gradient Boosting, Naive Bayes, and XGBoost, indicating that RCRI was a weaker comparator in this cohort (Supplementary Table S6; Supplementary Figure S3). Transparent Ridge coefficients are provided for interpretability in Supplementary Table S7, but Ridge was not treated as a prespecified primary model.

### 3.3. Benchmark Risk-Score Comparators

**Figure 4:**
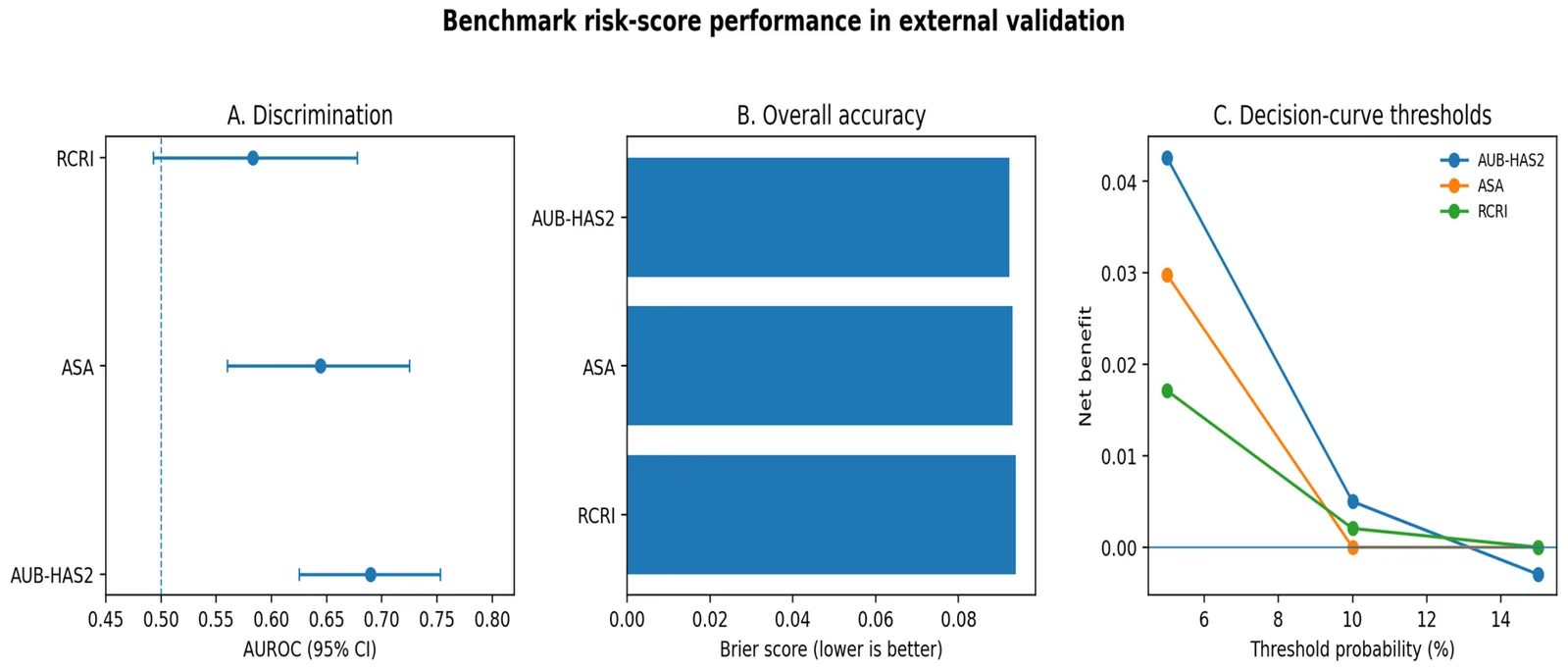
Benchmark risk-score performance in external validation. (**A**) Discrimination. Dots indicate the point estimate of the area under the receiver-operating-characteristic curve (AUROC) for each risk score; horizontal error bars show the 95% confidence interval. The vertical dashed line at AUROC = 0.50 marks the reference value for no discriminative ability (chance-level prediction). (**B**) Overall accuracy. Bars show the Brier score for each risk score (mean squared difference between predicted and observed outcomes); lower values indicate better overall accuracy. No lines or dots are used in this panel. (**C**) Decision-curve thresholds. Lines connect the net benefit of each risk score across a range of threshold probabilities (the probability at which a clinician would decide to intervene); dots mark the specific threshold values (5%, 10%, 15%) at which net benefit was evaluated. The solid horizontal line at net benefit = 0 represents the reference strategy of treating no patients. Overall and cohort-stratified benchmark score graphics are shown in Supplementary Figures S1 and S2, with full benchmark metrics in Supplementary Table S3.

Among benchmark scores, AUB-HAS2 had the highest AUROC. In the overall cohort, AUB-HAS2 AUROC was 0.759 (95% CI: 0.708–0.805), compared with 0.647 (95% CI: 0.580–0.708) for ASA and 0.587 (95% CI: 0.515–0.657) for RCRI (Table 3). In external validation, AUB-HAS2 had AUROC 0.690 (95% CI: 0.625–0.753), while ASA achieved AUROC 0.644 (95% CI: 0.560–0.725) and RCRI 0.583 (95% CI: 0.493–0.678). Calibration and decision-curve patterns reinforced the value of assessing more than AUROC. AUB-HAS2 had consistent discrimination and generally favorable net benefit at the 5% threshold, but calibration differed by cohort and threshold-specific utility narrowed at higher risk thresholds (Table 3; Figure 4).

## 4. Discussion

### 4.1. Principal Findings

In this two-center study, we developed and externally validated ten (ML) models for predicting 30-day MACE after noncardiac surgery and benchmarked their performance against established clinical risk scores. Three principal findings emerged. First, no ML algorithm consistently outperformed the others across all performance domains. Naive Bayes achieved the highest discrimination, whereas Gradient Boosting showed an approximately preserved calibration slope while still underestimating absolute risk externally (observed-to-expected ratio, 1.79), and HistGradient Boosting demonstrated the best overall probability prediction and decision-curve profile. Second, despite these metric-specific differences, no ML model demonstrated statistically significant superiority over the AUB-HAS2 score in external validation. Third, model rankings varied substantially depending on whether discrimination, calibration, prediction error, or clinical utility was evaluated, highlighting that reliance on AUROC alone may provide an incomplete and potentially misleading assessment of model performance.

Collectively, these findings suggest that both ML-based models and established clinical risk scores provide moderate rather than definitive risk stratification. They may assist clinicians in identifying patients who warrant closer perioperative evaluation or postoperative surveillance but should complement, rather than replace, comprehensive clinical assessment, including surgical risk, functional capacity, frailty, clinician judgment, and multidisciplinary decision-making.

### 4.2. Clinical Interpretation

Machine learning has generated considerable enthusiasm for perioperative risk prediction because of its ability to model complex nonlinear relationships and higher-order interactions among routinely collected clinical variables. However, our findings demonstrate that increased algorithmic complexity does not necessarily translate into clinically meaningful improvements after rigorous external validation. Although several ML algorithms achieved modest improvements in selected performance metrics, none consistently demonstrated superior discrimination, calibration, and clinical utility simultaneously.

This observation is particularly relevant because clinical implementation depends on accurate probability estimation rather than discrimination alone. Naive Bayes achieved the highest AUROC but exhibited suboptimal calibration and threshold-dependent decision-curve performance, indicating that good ranking ability did not translate into reliable, individualized risk estimates. In contrast, Gradient Boosting and HistGradient Boosting produced more balanced performance profiles, with more favorable calibration slopes and lower prediction error despite slightly lower AUROC values, although both still underestimated absolute risk in the external cohort. These findings emphasize that model selection should reflect the intended clinical application rather than a single summary statistic.

## 5. Comparison with Established Clinical Risk Scores

The strong performance of AUB-HAS2 deserves particular attention. Unlike ML algorithms, AUB-HAS2 relies exclusively on a small number of readily available clinical variables that capture major determinants of perioperative cardiovascular risk, including age, anemia, heart disease, cardiovascular symptoms, emergency surgery, and vascular procedures. Despite its simplicity, AUB-HAS2 remained highly competitive: no ML algorithm demonstrated statistically significant AUROC superiority over it in external validation, although the present design cannot establish formal non-inferiority. This finding suggests that carefully selected clinical predictors continue to capture much of the prognostic information required for perioperative cardiovascular risk assessment.

By contrast, RCRI and ASA demonstrated weaker performance in our cohort. This observation is consistent with previous studies suggesting that RCRI may underestimate risk in contemporary surgical populations with greater comorbidity burden and more heterogeneous operative profiles. Although these traditional scores remain clinically useful because of their simplicity and familiarity, our results suggest that AUB-HAS2 currently represents a stronger benchmark for contemporary perioperative cardiovascular risk prediction.

## 6. Implications for Clinical Artificial Intelligence

Our findings also contribute to the broader discussion regarding the clinical implementation of artificial intelligence. Numerous ML studies report excellent discrimination, yet many rely on internal validation or random train–test splits that may overestimate real-world performance. By deliberately separating model development and validation across independent institutions, the PERICARE study provides a more rigorous assessment of model transportability. The attenuation in performance observed after external validation reinforces growing evidence that algorithmic gains often diminish when models are applied to populations with different case mixes, practice patterns, and outcome prevalences. In the present study, these shifts were substantial and quantifiable: the external cohort was older (median age, 72 versus 65 years), had a threefold higher emergency-surgery burden (48.7% versus 15.8%), more frequently had functional capacity below 4 METs (32.4% versus 16.1%), and had a higher 30-day MACE prevalence (10.1% versus 3.8%). This combined shift in case mix and baseline risk plausibly explains why every model and benchmark score underestimated absolute risk in external validation (observed-to-expected ratios above 1; Figure 3) and illustrates why moderate discrimination does not guarantee transportable absolute-risk estimates. Rather than a model-specific deficiency, systematic underprediction of this kind is an expected consequence of transporting absolute risks across institutions with different referral patterns; intercept updating on a modest local sample would be the minimal recalibration step before any absolute-risk use.

Equally important, our results demonstrate that different evaluation metrics frequently favor different algorithms. Discrimination, calibration, prediction error, and clinical utility each measure distinct aspects of model performance and should therefore be interpreted together rather than hierarchically. In perioperative medicine, where decisions regarding additional testing, perioperative optimization, monitoring intensity, and postoperative troponin surveillance depend on estimated absolute risk, calibration and clinical utility are arguably as important as discrimination. Comprehensive model evaluation should therefore become standard practice in future perioperative ML research.

## 7. Methodological Considerations

Several methodological features strengthen the validity of the present study. First, model development and validation were intentionally performed in separate institutions rather than by random data partitioning, providing a realistic assessment of external transportability. Second, all candidate models were evaluated using multiple complementary performance metrics, including AUROC, calibration, Brier score, and decision-curve analysis, thereby avoiding overreliance on discrimination alone. Third, established clinical risk scores were included as benchmark comparators, enabling assessment of whether increased algorithmic complexity translated into meaningful clinical improvement. Finally, study reporting followed TRIPOD+AI recommendations, and risk of bias and applicability were systematically evaluated using PROBAST+AI, enhancing methodological transparency.

## 8. Limitations

Several limitations should be acknowledged. First, this was an observational two-center study, and residual confounding, documentation heterogeneity, and outcome ascertainment bias cannot be completely excluded. Second, the number of MACEs was relatively limited, resulting in wide confidence intervals and potentially unstable estimates for more complex ML algorithms. With 27 derivation events relative to the candidate predictor set, the risk of model instability and overfitting is substantial, particularly for flexible nonlinear algorithms; penalization, fixed conservative hyperparameters, and training-only preprocessing mitigate but cannot eliminate this limitation, and site-separated external validation assesses its consequences for transportability without correcting instability introduced during model development. Algorithm-specific estimates and model rankings should therefore be interpreted as exploratory. Third, although site-separated external validation provides a rigorous assessment of transportability, differences between institutions likely contributed to calibration drift. Fourth, cardiac biomarkers, including troponin and NT-proBNP, were unavailable for a substantial proportion of patients and therefore could not be incorporated into the primary prediction models. Finally, this study evaluated predictive performance rather than clinical impact. Whether implementation of these models improves patient outcomes, resource utilization, or clinical workflow should be determined through prospective implementation studies, including local recalibration and integration into clinician-facing decision-support systems.

## 9. Conclusions

In this two-center external validation study, machine-learning models achieved moderate performance for predicting 30-day major adverse cardiovascular events after noncardiac surgery, but no algorithm consistently outperformed established clinical risk scores across discrimination, calibration, prediction error, and clinical utility. Although Naive Bayes achieved the highest discrimination, Gradient Boosting showed the calibration slope closest to unity (although absolute risk remained underestimated externally), and HistGradient Boosting provided the best overall probability prediction, none showed statistically significant superiority over the AUB-HAS2 score. These findings suggest that rigorous external validation and comprehensive model evaluation are essential before ML models can be adopted for routine perioperative cardiovascular decision support. No evaluated model is sufficiently validated for routine clinical implementation or individual patient decision-making, and the ML analyses should be regarded as exploratory and hypothesis-generating. Future research should prioritize model transportability, local recalibration, prospective clinical impact evaluation, and integration into guideline-based perioperative care pathways.

## Figures and Tables

**Table 1:** Baseline characteristics of the total cohort, derivation cohort, external validation cohort, and MACE strata.

Characteristic	Overall	Derivation Cohort	External Validation Cohort	MACE−	MACE+
N	1085	707	378	1020	65
Age median [IQR]	68.0 [57.0–75.0]	65.0 [55.0–73.0]	72.0 [63.0–80.0]	67.0 [56.0–75.0]	76.5 [69.0–82.0]
Hemoglobin (g/dL) median [IQR]	12.1 [10.7–13.6]	12.4 [11.0–13.9]	11.6 [10.2–13.0]	12.2 [10.7–13.7]	11.0 [9.9–12.0]
Creatinine (mg/dL) median [IQR]	0.8 [0.7–1.1]	0.9 [0.7–1.1]	0.8 [0.7–1.1]	0.8 [0.7–1.1]	1.0 [0.7–1.4]
eGFR (mL/min/1.73 m^2^) median [IQR]	84.0 [62.0–96.0]	84.0 [62.0–97.0]	82.0 [60.0–95.0]	84.0 [63.0–96.0]	70.0 [43.0–92.0]
Diabetes Mellitus (DM) *n* (%)	379 (35.2%)	250 (35.7%)	129 (34.1%)	348 (34.4%)	31 (47.7%)
Hypertension (HT) *n* (%)	655 (60.7%)	409 (58.2%)	246 (65.4%)	609 (60.1%)	46 (70.8%)
Coronary Artery Disease (CAD) *n* (%)	358 (33.1%)	275 (39.1%)	83 (22.0%)	336 (33.1%)	22 (33.8%)
History of Myocardial Infarction (MI) *n* (%)	154 (14.2%)	127 (18.0%)	27 (7.1%)	143 (14.0%)	11 (16.9%)
History of Pre-op Heart Failure *n* (%)	50 (4.6%)	36 (5.1%)	14 (3.7%)	44 (4.3%)	6 (9.2%)
Chronic Kidney Disease (CKD) *n* (%)	99 (9.2%)	63 (9.0%)	36 (9.5%)	86 (8.5%)	13 (20.0%)
Cerebrovascular Disease (CVD) *n* (%)	94 (8.7%)	41 (5.8%)	53 (14.0%)	81 (8.0%)	13 (20.0%)
Peripheral Artery Disease (PAD) *n* (%)	41 (3.8%)	29 (4.1%)	12 (3.2%)	37 (3.6%)	4 (6.2%)
Atrial Fibrillation (AF) *n* (%)	104 (9.7%)	53 (7.6%)	51 (13.6%)	87 (8.6%)	17 (26.6%)
High-Risk Surgery *n* (%)	84 (7.7%)	64 (9.1%)	20 (5.3%)	81 (7.9%)	3 (4.6%)
Vascular Surgery *n* (%)	10 (0.9%)	10 (1.4%)	0 (0.0%)	9 (0.9%)	1 (1.5%)
Sex Female	552 (51.3%)	358 (50.6%)	194 (52.4%)	519 (51.2%)	33 (51.6%)
Sex Male	525 (48.7%)	349 (49.4%)	176 (47.6%)	494 (48.8%)	31 (48.4%)
Functional Capacity Assessment ≥ 4 MET	707 (66.8%)	510 (73.4%)	197 (54.1%)	689 (69.2%)	18 (28.6%)
Functional Capacity Assessment < 4 MET	230 (21.7%)	112 (16.1%)	118 (32.4%)	203 (20.4%)	27 (42.9%)
Functional Capacity Assessment Unclear	122 (11.5%)	73 (10.5%)	49 (13.5%)	104 (10.4%)	18 (28.6%)
ESC Surgical Risk Intermediate (1–5%)	693 (63.9%)	429 (60.7%)	264 (69.8%)	644 (63.1%)	49 (75.4%)
ESC Surgical Risk Low (<1%)	306 (28.2%)	239 (33.8%)	67 (17.7%)	294 (28.8%)	12 (18.5%)
ESC Surgical Risk High (>5%)	73 (6.7%)	32 (4.5%)	41 (10.8%)	70 (6.9%)	3 (4.6%)
ESC Surgical Risk Unknown	13 (1.2%)	7 (1.0%)	6 (1.6%)	12 (1.2%)	1 (1.5%)
Urgency of Surgery Elective	772 (72.7%)	581 (84.2%)	191 (51.3%)	743 (74.4%)	29 (45.3%)
Urgency of Surgery Emergency	290 (27.3%)	109 (15.8%)	181 (48.7%)	255 (25.6%)	35 (54.7%)
ASA 3	436 (40.2%)	263 (37.2%)	173 (45.8%)	410 (40.2%)	26 (40.0%)
ASA 4	285 (26.3%)	199 (28.1%)	86 (22.8%)	255 (25.0%)	30 (46.2%)
ASA 2	211 (19.4%)	150 (21.2%)	61 (16.1%)	206 (20.2%)	5 (7.7%)
ASA 1	153 (14.1%)	95 (13.4%)	58 (15.3%)	149 (14.6%)	4 (6.2%)
RCRI 0	571 (52.6%)	349 (49.4%)	222 (58.7%)	544 (53.3%)	27 (41.5%)
RCRI 1	377 (34.7%)	258 (36.5%)	119 (31.5%)	356 (34.9%)	21 (32.3%)
RCRI 2	114 (10.5%)	80 (11.3%)	34 (9.0%)	101 (9.9%)	13 (20.0%)
RCRI 3	19 (1.8%)	16 (2.3%)	3 (0.8%)	16 (1.6%)	3 (4.6%)
RCRI 4	4 (0.4%)	4 (0.6%)	0 (0.0%)	3 (0.3%)	1 (1.5%)
AUB-HAS2 1	321 (29.6%)	223 (31.5%)	98 (25.9%)	315 (30.9%)	6 (9.2%)
AUB-HAS2 2	295 (27.2%)	198 (28.0%)	97 (25.7%)	273 (26.8%)	22 (33.8%)
AUB-HAS2 0	227 (20.9%)	178 (25.2%)	49 (13.0%)	226 (22.2%)	1 (1.5%)
AUB-HAS2 3	168 (15.5%)	87 (12.3%)	81 (21.4%)	143 (14.0%)	25 (38.5%)
AUB-HAS2 4	63 (5.8%)	17 (2.4%)	46 (12.2%)	54 (5.3%)	9 (13.8%)
AUB-HAS2 5	11 (1.0%)	4 (0.6%)	7 (1.9%)	9 (0.9%)	2 (3.1%)

Abbreviations: ASA, American Society of Anesthesiologists physical status; AUB-HAS2, American University of Beirut-HAS2; RCRI, revised cardiac risk index. Note: Values are median [IQR] or *n* (%). Missingness is shown in-line when present. Full missingness by cohort is shown in Supplementary Table S1.

**Table 2:** External validation performance of clinical machine-learning models and benchmark scores.

Model/Score	Type	*n*/Events	AUROC (95% CI)	Brier	Cal. Slope	O:E	NB 5%	NB 10%	NB 15%
Ridge	model	378/38	0.683 (0.604–0.758)	0.091	0.749	1.744	0.046	0.010	0.001
Lasso	model	378/38	0.685 (0.607–0.761)	0.090	0.760	1.749	0.046	0.007	0.001
ElasticNet	model	378/38	0.677 (0.595–0.756)	0.093	0.957	2.270	0.024	0.001	0.001
Linear SVM	model	378/38	0.684 (0.606–0.758)	0.091	0.791	1.776	0.046	0.006	0.002
Random Forest	model	378/38	0.701 (0.623–0.775)	0.089	0.806	1.636	0.050	0.018	0.007
Gradient Boosting	model	378/38	0.707 (0.623–0.783)	0.088	0.991	1.791	0.045	0.015	0.011
HistGradient Boosting	model	378/38	0.694 (0.613–0.772)	0.087	0.813	1.628	0.048	0.027	0.011
AdaBoost	model	378/38	0.669 (0.576–0.750)	0.089	0.846	1.691	0.050	0.019	0.010
Naive Bayes	model	378/38	0.738 (0.668–0.804)	0.090	2.139	2.033	0.068	0.000	0.000
XGBoost	model	378/38	0.697 (0.617–0.769)	0.089	0.602	1.674	0.027	0.012	0.008
AUB-HAS2	benchmark	378/38	0.690 (0.625–0.753)	0.092	0.557	1.462	0.043	0.005	−0.003
ASA	benchmark	378/38	0.644 (0.560–0.725)	0.093	0.994	2.723	0.030	0.000	0.000
RCRI	benchmark	378/38	0.583 (0.493–0.678)	0.094	0.879	2.963	0.017	0.002	0.000

Abbreviations: ASA, American Society of Anesthesiologists physical status; AUB-HAS2, American University of Beirut-HAS2 index; AUROC: area under the receiver operating characteristic curve; NB, net benefit; RCRI, revised cardiac risk index. Note: No ML model was prespecified as primary. Models are interpreted across discrimination, probability accuracy, calibration, and decision-curve metrics. NB indicates decision-curve net benefit.

**Table 3:** Benchmark risk-score performance for 30-day MACE.

Score	Dataset	*n*/Events	AUROC (95% CI)	Brier	Cal. Slope	O: E	NB 5%	NB 10%	NB 15%
ASA	Overall	1085/65	0.647 (0.580–0.708)	0.056	0.964	1.587	0.015	0.000	0.000
ASA	Derivation cohort	707/27	0.664 (0.556–0.759)	0.036	1.061	1.000	0.008	0.000	0.000
ASA	External validation cohort	378/38	0.644 (0.560–0.725)	0.093	0.994	2.723	0.030	0.000	0.000
RCRI	Overall	1085/65	0.587 (0.515–0.657)	0.056	0.794	1.632	0.010	0.002	0.000
RCRI	Derivation cohort	707/27	0.626 (0.509–0.739)	0.036	1.039	1.000	0.006	0.002	0.001
RCRI	External validation cohort	378/38	0.583 (0.493–0.678)	0.094	0.879	2.963	0.017	0.002	0.000
AUB-HAS2	Overall	1085/65	0.759 (0.708–0.805)	0.055	0.829	1.227	0.023	0.004	−0.000
AUB-HAS2	Derivation cohort	707/27	0.768 (0.677–0.854)	0.035	1.031	1.000	0.013	0.003	0.001
AUB-HAS2	External validation cohort	378/38	0.690 (0.625–0.753)	0.092	0.557	1.462	0.043	0.005	−0.003

Abbreviations: ASA, American Society of Anesthesiologists physical status; AUB-HAS2, American University of Beirut-HAS2; AUROC, area under the receiver operating characteristic curve; NB, net benefit; RCRI, revised cardiac risk index. Note: AUB-HAS2, ASA, and RCRI were evaluated overall and by analytic cohort. NB indicates decision-curve net benefit at the specified threshold probability.

## Data Availability

The datasets presented in this article are not readily available because of regulations preventing data to be shared with anyone other than the researchers. A tool to use our algorithm is published on https://www.pericaremace.com/as open access. Requests to access the datasets should be directed to corresponding author.

## Supplementary Materials

The following supporting information can be downloaded at https://www.mdpi.com/article/10.3390/jcm15176848/s1, Table S1: Missingness by cohort; Table S2: Full baseline characteristics; Table S3: Full benchmark risk-score performance by cohort; Table S4: Clinical model performance in development cross-validation and external validation; Table S5: All clinical models and benchmark scores in external validation; Table S6: Paired bootstrap comparisons of each clinical model versus benchmark scores in external validation; Table S7: Transparent Ridge coefficients; Table S8: Algorithm implementation and hyperparameter specification; Figure S1: Benchmark risk-score performance in the overall cohort; Figure S2: Benchmark risk-score performance across the overall, derivation, and external validation cohorts; Figure S3: Paired external validation comparisons of clinical ML models with benchmark scores; Figure S4: Per-model external validation overview for clinical ML models.

## References

[1] Thompson A., Fleischmann K.E., Smilowitz N.R., de Las Fuentes L., Mukherjee D., Aggarwal N.R., Ahmad F.S., Allen R.B., Altin S.E., Auerbach A. (2024). 2024 AHA/ACC/ACS/ASNC/HRS/SCA/SCCT/SCMR/SVM Guideline for perioperative cardiovascular management for noncardiac surgery. J. Am. Coll. Cardiol..

[2] Halvorsen S., Mehilli J., Cassese S., Hall T.S., Abdelhamid M., Barbato E., De Hert S., de Laval I., Geisler T., Hinterbuchner L. (2022). 2022 ESC Guidelines on cardiovascular assessment and management of patients undergoing non-cardiac surgery. Eur. Heart J..

[3] Lee T.H., Marcantonio E.R., Mangione C.M., Thomas E.J., Polanczyk C.A., Cook E.F., Sugarbaker D.J., Donaldson M.C., Poss R., Ho K.K.L. (1999). Derivation and prospective validation of a simple index for prediction of cardiac risk of major noncardiac surgery. Circulation.

[4] Sankar A., Johnson S.R., Beattie W.S., Tait G., Wijeysundera D.N. (2014). Reliability of the American society of anesthesiologists’ physical status scale in clinical practice. Br. J. Anaesth..

[5] Dakik H.A., Chehab O., Eldirani M., Sbeity E., Karam C., Abou Hassan O., Msheik M., Hassan H., Msheik A., Kaspar C. (2019). A new index for pre-operative cardiovascular evaluation. J. Am. Coll. Cardiol..

[6] Dakik H.A., Sbaity E., Msheik A., Kaspar C., Eldirani M., Chehab O., Abou Hassan O., Mailhac A., Makki M., Tamim H. (2020). AUB-HAS2 Cardiovascular risk index: Performance in surgical subpopulations and comparison to the revised cardiac risk index. J. Am. Heart Assoc..

[7] Armoundas A.A., Narayan S.M., Arnett D.K., Spector-Bagdady K., Bennett D.A., Celi L.A., Friedman P.A., Gollob M.H., Hall J.L., Kwitek A.E. (2024). Use of artificial intelligence in improving outcomes in heart disease: A scientific statement from the American Heart Association. Circulation.

[8] Christodoulou E., Ma J., Collins G.S., Steyerberg E.W., Verbakel J.Y., Van Calster B. (2019). A systematic review shows no performance benefit of machine learning over logistic regression for clinical prediction models. J. Clin. Epidemiol..

[9] Van Calster B., McLernon D.J., van Smeden M., Wynants L., Steyerberg E.W. (2019). Calibration: The Achilles heel of predictive analytics. BMC Med..

[10] Vickers A.J., Elkin E.B. (2006). Decision curve analysis: A novel method for evaluating prediction models. Med. Decis. Mak..

[11] Ito H., Seki T., Kawazoe Y., Takiguchi T., Akagi Y., Kubota K., Miyake K., Okada M., Ohe K. (2025). Evaluation of perioperative cardiovascular event risk in gastrointestinal surgery-predictive modeling and risk stratification using machine learning. Circ. J..

[12] Ren Y., Adiyeke E., Guan Z., Hu Z., Loftus T.J., Shickel B., Rashidi P., Ozrazgat-Baslanti T., Bihorac A. (2026). MySurgeryRisk model predictions of postoperative complications and mortality. JAMA Surg..

[13] Collins G.S., Moons K.G.M., Dhiman P., Riley R.D., Beam A.L., Van Calster B., Ghassemi M., Liu X., Reitsma J.B., van Smeden M. (2024). TRIPOD+AI statement: Updated guidance for reporting clinical prediction models that use regression or machine learning methods. BMJ.

[14] Moons K.G.M., Damen J.A.A., Kaul T., Hooft L., Andaur Navarro C., Dhiman P., Beam A.L., Van Calster B., Celi L.A., Denaxas S. (2025). PROBAST+AI: A tool to assess risk of bias and applicability of prediction model studies centered on artificial intelligence. BMJ..

[15] Al-Khatib S.M., Stevenson W.G., Ackerman M.J., Bryant W.J., Callans D.J., Curtis A.B., Deal B.J., Dickfeld T., Field M.E., Fonarow G.C. (2018). AHA/ACC/HRS guideline for management of patients with ventricular arrhythmias and the prevention of sudden cardiac death: Executive summary: A report of the American college of cardiology/American heart association task force on clinical practice guidelines and the heart rhythm society. Circulation.

[16] Thygesen K., Alpert J.S., Jaffe A.S., Chaitman B.R., Bax J.J., Morrow D.A., White H.D. (2018). Fourth Universal Definition of Myocardial Infarction (2018). Circulation.

[17] Hicks K.A., Mahaffey K.W., Mehran R., Nissen S.E., Wiviott S.D., Dunn B., Solomon S.D., Marler J.R., Teerlink J.R., Farb A. (2018). 2017 Cardiovascular and Stroke Endpoint Definitions for Clinical Trials. Circulation.

[18] Harrell FEJr Lee K.L., Mark D.B. (1996). Multivariable prognostic models: Issues in developing models, evaluating assumptions and adequacy, and measuring and reducing errors. Stat. Med..

[19] Steyerberg E.W. (2019). Clinical Prediction Models: A Practical Approach to Development, Validation, and Updating.

[20] Pedregosa F., Varoquaux G., Gramfort A., Michel V., Thirion B., Grisel O., Blondel M., Prettenhofer P., Weiss R., Dubourg V. (2011). Scikit-learn: Machine learning in Python. J. Mach. Learn. Res..

[21] Breiman L. (2001). Random forests. Mach. Learn..

[22] Chen T., Guestrin C. XGBoost: A scalable tree boosting system. Proceedings of the 22nd ACM SIGKDD International Conference on Knowledge Discovery and Data Mining.

[23] Brier G.W. (1950). Verification of forecasts expressed in terms of probability. Mon. Weather Rev..

[24] Van Calster B., Nieboer D., Vergouwe Y., De Cock B., Pencina M.J., Steyerberg E.W. (2016). A calibration hierarchy for risk models was defined: From utopia to empirical data. J. Clin. Epidemiol..

[25] DeLong E.R., DeLong D.M., Clarke-Pearson D.L. (1988). Comparing the areas under two or more correlated receiver operating characteristic curves: A nonparametric approach. Biometrics.

[26] Pencina M.J., D’Agostino R.B., D’Agostino R.B., Vasan R.S. (2008). Evaluating the added predictive ability of a new marker: From area under the ROC curve to reclassification and beyond. Stat. Med..

[27] Devereaux P.J., Chan M.T.V., Alonso-Coello P., Walsh M., Berwanger O., Villar J.C., Wang C.Y., Garutti R.I., Jacka M.J., Sigamani A. (2012). VISION study investigators. Association between postoperative troponin levels and 30-day mortality among patients undergoing noncardiac surgery. JAMA.

[28] Devereaux P.J., Szczeklik W. (2020). Myocardial injury after non-cardiac surgery. Eur. Heart J..

