# Supplementary material for: External Validation of Clinical Risk Scores and Machine Learning Models for Predicting 30-Day Cardiovascular Risk After Noncardiac Surgery: The PERICARE Study
Source: J Clin Med. 2026 Sep 4;15(17):6848. doi: 10.3390/jcm15176848 (PMC13566043; doi:10.3390/jcm15176848)
Supplement: Supplementary file 1 [file jcm-15-06848-s001.zip › jcm-4459478-supplementary.pdf]

Supplementary Materials

External Validation of Clinical Risk Scores and Machine Learning Models for Predicting 30-Day Cardiovascular Risk After Noncardiac Surgery: The PERICARE Study

This supplemental appendix contains expanded methods, supplemental tables, and supplemental figures. All supplemental tables and figures are cited in the main manuscript.

Clinical ML and statistical details

Clinical models used derivation-cohort preprocessing and model fitting, followed by external validation without refitting. Ten algorithms were evaluated without prespecifying a single primary ML model: ridge logistic regression, lasso logistic regression, elastic-net logistic regression, linear SVM, random forest, gradient boosting, histogram gradient boosting, AdaBoost, Naive Bayes, and XGBoost. Ridge coefficients are reported only to illustrate transparent model-scale effects; they do not imply that Ridge was the primary or best-performing model.

Bootstrap confidence intervals were used for discrimination, Brier score, and decision-curve net benefit. Paired bootstrap comparisons were used for model-versus-benchmark contrasts in the external validation cohort.

Supplemental Tables

Supplemental Table S1. Missingness by cohort.

Variable	Missing overall, %	Missing derivation, %	Missing external validation, %
NT_proBNP	94.1	91.9	98.1
Troponin I/T	89.2	86.7	93.9
eGFR (mL/min/1.73m²)	7.2	4.8	11.6
Creatinine (mg/dL)	2.8	3.5	1.3
Functional Capacity Assessment	2.4	1.7	3.7
Hemoglobin (g/dL)	2.0	2.3	1.6
Type of Surgery	1.1	0.8	1.6
Sex	0.7	0.0	2.1
Atrial Fibrillation (AF)	0.7	0.8	0.5
Diabetes Mellitus (DM)	0.6	1.0	0.0
Hypertension (HT)	0.6	0.6	0.5
Cerebrovascular Disease (SVH)	0.6	0.8	0.0
Peripheral Artery Disease (PAH)	0.5	0.6	0.3
Chronic Kidney Disease (KBH)	0.5	0.6	0.3
Coronary Artery Disease (KAH)	0.4	0.6	0.0
Age	0.2	0.0	0.5
Vascular Surgery	0.0	0.0	0.0
High-Risk Surgery	0.0	0.0	0.0
ESC Surgical Risk	0.0	0.0	0.0
History of Pre-op Heart Failure	0.0	0.0	0.0
History of Myocardial Infarction (MI)	0.0	0.0	0.0
Insulin-Treated DM	0.0	0.0	0.0
ASA	0.0	0.0	0.0
RCRI	0.0	0.0	0.0
AUB-HAS2	0.0	0.0	0.0

Note: Variables are ordered by missingness in the overall cohort. Abbreviations: ASA,American Society of Anesthesiologists physical status; AUB-HAS2 ,American University of Beirut-HAS2 ;RCRI, revised cardiac risk index

Supplemental Table S2. Full baseline characteristics.

Characteristic	Overall	Derivation cohort	External validation cohort	MACE-	MACE+
N	1085	707	378	1020	65
Age — median [IQR]	68.0 [57.0–75.0]	65.0 [55.0–73.0]	72.0 [63.0–80.0]	67.0 [56.0–75.0]	76.5 [69.0–82.0]
Hemoglobin (g/dL) median [IQR]	12.1 [10.7–13.6]	12.4 [11.0–13.9]	11.6 [10.2–13.0]	12.2 [10.7–13.7]	11.0 [9.9–12.0]
Creatinine (mg/dL) median [IQR]	0.8 [0.7–1.1]	0.9 [0.7–1.1]	0.8 [0.7–1.1]	0.8 [0.7–1.1]	1.0 [0.7–1.4]
eGFR (mL/min/1.73m²) median [IQR]	84.0 [62.0–96.0]	84.0 [62.0–97.0]	82.0 [60.0–95.0]	84.0 [63.0–96.0]	70.0 [43.0–92.0]
Pre-op LVEF (%) median [IQR]	60.0 [55.0–60.0]	60.0 [55.0–60.0]	60.0 [60.0–60.0]	60.0 [55.0–60.0]	60.0 [55.0–60.0]
Troponin I/T median [IQR]	13.0 [7.0–27.0]	12.3 [7.2–27.0]	17.0 [4.5–30.0]	12.0 [7.0–26.0]	37.0 [16.5–90.0]
NT_proBNP median [IQR]	407.5 [74.8–1876.2]	327.0 [74.0–1689.0]	2268.0 [989.5–5227.0]	279.0 [69.5–1294.0]	3700.0 [1700.0–10418.0]
Diabetes Mellitus (DM) n (%)	379 (35.2%)	250 (35.7%)	129 (34.1%)	348 (34.4%)	31 (47.7%)
Insulin-Treated DM n (%)	38 (3.5%)	27 (3.8%)	11 (2.9%)	33 (3.2%)	5 (7.7%)
Hypertension (HT) n (%)	655 (60.7%)	409 (58.2%)	246 (65.4%)	609 (60.1%)	46 (70.8%)
Coronary Artery Disease (KAH) n (%)	358 (33.1%)	275 (39.1%)	83 (22.0%)	336 (33.1%)	22 (33.8%)
History of Myocardial Infarction (MI) n (%)	154 (14.2%)	127 (18.0%)	27 (7.1%)	143 (14.0%)	11 (16.9%)
History of Pre-op Heart Failure n (%)	50 (4.6%)	36 (5.1%)	14 (3.7%)	44 (4.3%)	6 (9.2%)
Chronic Kidney Disease (KBH) n (%)	99 (9.2%)	63 (9.0%)	36 (9.5%)	86 (8.5%)	13 (20.0%)
Cerebrovascular Disease (SVH) n (%)	94 (8.7%)	41 (5.8%)	53 (14.0%)	81 (8.0%)	13 (20.0%)
Peripheral Artery Disease (PAH) n (%)	41 (3.8%)	29 (4.1%)	12 (3.2%)	37 (3.6%)	4 (6.2%)
Atrial Fibrillation (AF) n (%)	104 (9.7%)	53 (7.6%)	51 (13.6%)	87 (8.6%)	17 (26.6%)
High-Risk Surgery n (%)	84 (7.7%)	64 (9.1%)	20 (5.3%)	81 (7.9%)	3 (4.6%)
Vascular Surgery n (%)	10 (0.9%)	10 (1.4%)	0 (0.0%)	9 (0.9%)	1 (1.5%)
Sex Female	552 (51.3%)	358 (50.6%)	194 (52.4%)	519 (51.2%)	33 (51.6%)
Sex Male	525 (48.7%)	349 (49.4%)	176 (47.6%)	494 (48.8%)	31 (48.4%)
Functional Capacity Assessment ≥4 MET	707 (66.8%)	510 (73.4%)	197 (54.1%)	689 (69.2%)	18 (28.6%)
Functional Capacity Assessment <4 MET	230 (21.7%)	112 (16.1%)	118 (32.4%)	203 (20.4%)	27 (42.9%)
Functional Capacity Assessment Unclear / not assessable	122 (11.5%)	73 (10.5%)	49 (13.5%)	104 (10.4%)	18 (28.6%)
ESC Surgical Risk Intermediate (1–5%)	693 (63.9%)	429 (60.7%)	264 (69.8%)	644 (63.1%)	49 (75.4%)
ESC Surgical Risk Low (<1%)	306 (28.2%)	239 (33.8%)	67 (17.7%)	294 (28.8%)	12 (18.5%)

Characteristic	Overall	Derivation cohort	External validation cohort	MACE-	MACE+
ESC Surgical Risk High (>5%)	73 (6.7%)	32 (4.5%)	41 (10.8%)	70 (6.9%)	3 (4.6%)
ESC Surgical Risk Unknown	13 (1.2%)	7 (1.0%)	6 (1.6%)	12 (1.2%)	1 (1.5%)
Urgency of Surgery Elective	772 (72.7%)	581 (84.2%)	191 (51.3%)	743 (74.4%)	29 (45.3%)
Urgency of Surgery Emergency	290 (27.3%)	109 (15.8%)	181 (48.7%)	255 (25.6%)	35 (54.7%)
Type of Surgery General Surgery	112 (10.4%)	111 (15.8%)	1 (0.3%)	105 (10.4%)	7 (10.8%)
Type of Surgery Orthopedics	73 (6.8%)	66 (9.4%)	7 (1.9%)	71 (7.0%)	2 (3.1%)
Type of Surgery Urology	72 (6.7%)	71 (10.1%)	1 (0.3%)	72 (7.1%)	0 (0.0%)
Type of Surgery Obstetrics	58 (5.4%)	56 (8.0%)	2 (0.5%)	58 (5.8%)	0 (0.0%)
Type of Surgery Ophthalmology	40 (3.7%)	40 (5.7%)	0 (0.0%)	40 (4.0%)	0 (0.0%)
Type of Surgery Obstetrics and Gynecology	33 (3.1%)	33 (4.7%)	0 (0.0%)	32 (3.2%)	1 (1.5%)
Type of Surgery Thoracic Surgery	32 (3.0%)	32 (4.6%)	0 (0.0%)	32 (3.2%)	0 (0.0%)
Type of Surgery Orthopedics	25 (2.3%)	25 (3.6%)	0 (0.0%)	20 (2.0%)	5 (7.7%)
ASA 3	436 (40.2%)	263 (37.2%)	173 (45.8%)	410 (40.2%)	26 (40.0%)
ASA 4	285 (26.3%)	199 (28.1%)	86 (22.8%)	255 (25.0%)	30 (46.2%)
ASA 2	211 (19.4%)	150 (21.2%)	61 (16.1%)	206 (20.2%)	5 (7.7%)
ASA 1	153 (14.1%)	95 (13.4%)	58 (15.3%)	149 (14.6%)	4 (6.2%)
RCRI 0	571 (52.6%)	349 (49.4%)	222 (58.7%)	544 (53.3%)	27 (41.5%)
RCRI 1	377 (34.7%)	258 (36.5%)	119 (31.5%)	356 (34.9%)	21 (32.3%)
RCRI 2	114 (10.5%)	80 (11.3%)	34 (9.0%)	101 (9.9%)	13 (20.0%)
RCRI 3	19 (1.8%)	16 (2.3%)	3 (0.8%)	16 (1.6%)	3 (4.6%)
RCRI 4	4 (0.4%)	4 (0.6%)	0 (0.0%)	3 (0.3%)	1 (1.5%)
AUB-HAS2 1	321 (29.6%)	223 (31.5%)	98 (25.9%)	315 (30.9%)	6 (9.2%)
AUB-HAS2 2	295 (27.2%)	198 (28.0%)	97 (25.7%)	273 (26.8%)	22 (33.8%)
AUB-HAS2 0	227 (20.9%)	178 (25.2%)	49 (13.0%)	226 (22.2%)	1 (1.5%)
AUB-HAS2 3	168 (15.5%)	87 (12.3%)	81 (21.4%)	143 (14.0%)	25 (38.5%)
AUB-HAS2 4	63 (5.8%)	17 (2.4%)	46 (12.2%)	54 (5.3%)	9 (13.8%)
AUB-HAS2 5	11 (1.0%)	4 (0.6%)	7 (1.9%)	9 (0.9%)	2 (3.1%)

Note: Same content as the complete baseline table used for modeling interpretation. Abbreviations: ASA,American Society of Anesthesiologists physical status; AUB-HAS2 ,American University of Beirut-HAS2 ;RCRI, revised cardiac risk index

Supplemental Table S3. Full benchmark risk-score performance by cohort.

Score	Dataset	n/events	AUROC (95% CI)	Brier	Cal. slope	O:E	NB 5%	NB 10%	NB 15%
ASA	Overall	1085/65	0.647 (0.580–0.708)	0.056	0.964	1.587	0.015	0.000	0.000
ASA	Derivation cohort	707/27	0.664 (0.556–0.759)	0.036	1.061	1.000	0.008	0.000	0.000
ASA	External validation cohort	378/38	0.644 (0.560–0.725)	0.093	0.994	2.723	0.030	0.000	0.000
RCRI	Overall	1085/65	0.587 (0.515–0.657)	0.056	0.794	1.632	0.010	0.002	0.000
RCRI	Derivation cohort	707/27	0.626 (0.509–0.739)	0.036	1.039	1.000	0.006	0.002	0.001
RCRI	External validation cohort	378/38	0.583 (0.493–0.678)	0.094	0.879	2.963	0.017	0.002	0.000
AUB-HAS2	Overall	1085/65	0.759 (0.708–0.805)	0.055	0.829	1.227	0.023	0.004	-0.000
AUB-HAS2	Derivation cohort	707/27	0.768 (0.677–0.854)	0.035	1.031	1.000	0.013	0.003	0.001
AUB-HAS2	External validation cohort	378/38	0.690 (0.625–0.753)	0.092	0.557	1.462	0.043	0.005	-0.003

Note: Performance shown overall and by analytic cohort. Abbreviations: ASA,American Society of Anesthesiologists physical status; AUB-HAS2 ,American University of Beirut-HAS2 ;RCRI, revised cardiac risk index

Supplemental Table S4. Clinical model performance in development cross-validation and external validation.

Model	Validation	n/events	AUROC (95% CI)	Brier (95% CI)	IPA	Cal. slope	Cal. intercept	O:E	NB 5%	NB 10%	NB 15%
Ridge	Derivation internal CV	707/27	0.768 (0.670–0.855)	0.035 (0.024–0.048)	0.040	1.275	-0.076	0.932	0.011	0.005	0.000
Ridge	External validation	378/38	0.683 (0.604–0.758)	0.091 (0.066–0.117)	-0.002	0.749	0.636	1.744	0.046	0.010	0.001
Lasso	Derivation internal CV	707/27	0.765 (0.668–0.853)	0.035 (0.024–0.048)	0.039	1.267	-0.075	0.932	0.012	0.006	0.000
Lasso	External validation	378/38	0.685 (0.607–0.761)	0.090 (0.066–0.117)	-0.000	0.760	0.639	1.749	0.046	0.007	0.001
ElasticNet	Derivation internal CV	707/27	0.738 (0.631–	0.037 (0.025–	-0.001	1.099	-0.095	0.914	0.012	0.000	-0.002

Model	Validation	n/events	AUROC (95% CI)	Brier (95% CI)	IPA	Cal. slope	Cal. intercept	O:E	NB 5%	NB 10%	NB 15%
			0.832)	0.051)							
ElasticNet	External validation	378/38	0.677 (0.595–0.756)	0.093 (0.067–0.121)	-0.031	0.957	0.901	2.270	0.024	0.001	0.001
Linear SVM	Derivation internal CV	707/27	0.760 (0.663–0.848)	0.036 (0.024–0.049)	0.032	1.223	-0.076	0.932	0.011	0.004	0.001
Linear SVM	External validation	378/38	0.684 (0.606–0.758)	0.091 (0.066–0.117)	-0.002	0.791	0.653	1.776	0.046	0.006	0.002
Random Forest	Derivation internal CV	707/27	0.756 (0.659–0.843)	0.036 (0.024–0.049)	0.032	1.253	-0.070	0.936	0.012	0.002	-0.002
Random Forest	External validation	378/38	0.701 (0.623–0.775)	0.089 (0.064–0.115)	0.019	0.806	0.569	1.636	0.050	0.018	0.007
Gradient Boosting	Derivation internal CV	707/27	0.714 (0.620–0.802)	0.036 (0.024–0.050)	0.016	1.334	-0.081	0.926	0.007	0.001	-0.001
Gradient Boosting	External validation	378/38	0.707 (0.623–0.783)	0.088 (0.064–0.114)	0.025	0.991	0.667	1.791	0.045	0.015	0.011
HistGradient Boosting	Derivation internal CV	707/27	0.750 (0.646–0.842)	0.035 (0.024–0.049)	0.035	1.405	-0.079	0.928	0.011	0.001	-0.000
HistGradient Boosting	External validation	378/38	0.694 (0.613–0.772)	0.087 (0.063–0.113)	0.033	0.813	0.567	1.628	0.048	0.027	0.011
AdaBoost	Derivation internal CV	707/27	0.762 (0.658–0.848)	0.035 (0.024–0.048)	0.041	1.488	-0.084	0.924	0.010	0.007	0.001
AdaBoost	External validation	378/38	0.669 (0.576–0.750)	0.089 (0.065–0.115)	0.015	0.846	0.595	1.691	0.050	0.019	0.010
Naive Bayes	Derivation internal CV	707/27	0.766 (0.671–0.845)	0.036 (0.024–0.050)	0.026	2.059	-0.065	0.940	0.011	0.000	0.000
Naive Bayes	External validation	378/38	0.738 (0.668–0.804)	0.090 (0.065–0.118)	0.001	2.139	0.778	2.033	0.068	0.000	0.000
XGBoost	Derivation internal CV	707/27	0.719 (0.592–0.837)	0.036 (0.024–0.049)	0.024	1.088	-0.138	0.880	0.012	0.001	0.001
XGBoost	External validation	378/38	0.697 (0.617–0.769)	0.089 (0.064–0.116)	0.010	0.602	0.643	1.674	0.027	0.012	0.008

Note: All 10 ML models were evaluated without a prespecified primary model.

Supplemental Table S5. All clinical models and benchmark scores in external validation.

Model / score	Type	n/events	AUROC (95% CI)	Brier	Cal. slope	O:E	NB 5%	NB 10%	NB 15%
Ridge	model	378/38	0.683 (0.604–0.758)	0.091	0.749	1.744	0.046	0.010	0.001
Lasso	model	378/38	0.685 (0.607–0.761)	0.090	0.760	1.749	0.046	0.007	0.001
ElasticNet	model	378/38	0.677 (0.595–0.756)	0.093	0.957	2.270	0.024	0.001	0.001
Linear SVM	model	378/38	0.684 (0.606–0.758)	0.091	0.791	1.776	0.046	0.006	0.002
Random Forest	model	378/38	0.701 (0.623–0.775)	0.089	0.806	1.636	0.050	0.018	0.007
Gradient Boosting	model	378/38	0.707 (0.623–0.783)	0.088	0.991	1.791	0.045	0.015	0.011
HistGradient Boosting	model	378/38	0.694 (0.613–0.772)	0.087	0.813	1.628	0.048	0.027	0.011
AdaBoost	model	378/38	0.669 (0.576–0.750)	0.089	0.846	1.691	0.050	0.019	0.010
Naive Bayes	model	378/38	0.738 (0.668–0.804)	0.090	2.139	2.033	0.068	0.000	0.000
XGBoost	model	378/38	0.697 (0.617–0.769)	0.089	0.602	1.674	0.027	0.012	0.008
AUB-HAS2	benchmark	378/38	0.690 (0.625–0.753)	0.092	0.557	1.462	0.043	0.005	-0.003
ASA	benchmark	378/38	0.644 (0.560–0.725)	0.093	0.994	2.723	0.030	0.000	0.000
RCRI	benchmark	378/38	0.583 (0.493–	0.094	0.879	2.963	0.017	0.002	0.000

Model / score	Type	n/events	AUROC (95% CI)	Brier	Cal. slope	O:E	NB 5%	NB 10%	NB 15%
			0.678)						

Note: This table supports the external validation comparisons in the main manuscript. Abbreviations: ASA,American Society of Anesthesiologists physical status; AUB-HAS2 ,American University of Beirut-HAS2 ;RCRI, revised cardiac risk index

Supplemental Table S6. Paired bootstrap comparisons of each clinical model versus benchmark scores in external validation.

Model	Benchmark	ΔAUROC	ΔAUROC lo	ΔAUROC hi	P	ΔBrier	ΔIPA	ΔNB 5%	ΔNB 10%	ΔNB 15%
Ridge	AUB-HAS2	-0.007	-0.071	0.058	0.800	-0.002	0.019	0.004	0.005	0.004
Ridge	ASA	0.038	-0.061	0.135	0.453	-0.002	0.027	0.016	0.010	0.001
Ridge	RCRI	0.099	-0.020	0.215	0.108	-0.003	0.036	0.029	0.008	0.001
Lasso	AUB-HAS2	-0.005	-0.068	0.060	0.855	-0.002	0.020	0.004	0.002	0.004
Lasso	ASA	0.041	-0.057	0.136	0.414	-0.003	0.028	0.017	0.007	0.001
Lasso	RCRI	0.102	-0.016	0.217	0.097	-0.003	0.038	0.029	0.005	0.001
ElasticNet	AUB-HAS2	-0.013	-0.088	0.067	0.764	0.001	-0.010	-0.019	-0.004	0.004
ElasticNet	ASA	0.033	-0.076	0.146	0.545	0.000	-0.003	-0.006	0.001	0.001
ElasticNet	RCRI	0.094	-0.031	0.219	0.139	-0.001	0.007	0.006	-0.001	0.001
Linear SVM	AUB-HAS2	-0.006	-0.068	0.058	0.831	-0.002	0.019	0.004	0.001	0.005
Linear SVM	ASA	0.039	-0.058	0.135	0.430	-0.002	0.026	0.017	0.006	0.002
Linear SVM	RCRI	0.100	-0.019	0.216	0.106	-0.003	0.036	0.029	0.004	0.002
Random Forest	AUB-HAS2	0.011	-0.053	0.075	0.719	-0.004	0.039	0.007	0.013	0.010
Random Forest	ASA	0.057	-0.035	0.149	0.234	-0.004	0.047	0.020	0.018	0.007
Random Forest	RCRI	0.118	-0.002	0.233	0.053	-0.005	0.057	0.033	0.016	0.007
Gradient Boosting	AUB-HAS2	0.017	-0.047	0.083	0.600	-0.004	0.046	0.002	0.010	0.014
Gradient Boosting	ASA	0.063	-0.031	0.155	0.195	-0.005	0.054	0.015	0.015	0.011
Gradient Boosting	RCRI	0.124	0.010	0.229	0.035	-0.006	0.064	0.028	0.013	0.011
HistGradient Boosting	AUB-HAS2	0.004	-0.056	0.065	0.904	-0.005	0.054	0.005	0.022	0.014
HistGradient Boosting	ASA	0.049	-0.047	0.144	0.308	-0.006	0.061	0.018	0.027	0.011
HistGradient Boosting	RCRI	0.111	-0.003	0.219	0.057	-0.006	0.071	0.030	0.025	0.011
AdaBoost	AUB-HAS2	-0.021	-0.095	0.047	0.527	-0.003	0.036	0.007	0.014	0.013
AdaBoost	ASA	0.025	-0.086	0.130	0.638	-0.004	0.043	0.020	0.019	0.010
AdaBoost	RCRI	0.086	-0.043	0.206	0.188	-0.005	0.053	0.032	0.017	0.010
Naive Bayes	AUB-HAS2	0.048	-0.027	0.126	0.235	-0.002	0.022	0.025	-0.005	0.003
Naive Bayes	ASA	0.093	0.022	0.176	0.009	-0.003	0.030	0.038	0.000	0.000
Naive Bayes	RCRI	0.154	0.050	0.256	0.004	-0.004	0.039	0.051	-0.002	0.000

Model	Benchmark	ΔAUROC	ΔAUROC lo	ΔAUROC hi	P	ΔBrier	ΔIPA	ΔNB 5%	ΔNB 10%	ΔNB 15%
XGBoost	AUB-HAS2	0.007	-0.057	0.070	0.842	-0.003	0.031	-0.015	0.007	0.011
XGBoost	ASA	0.052	-0.043	0.147	0.281	-0.004	0.039	-0.003	0.012	0.008
XGBoost	RCRI	0.113	0.001	0.223	0.048	-0.004	0.048	0.010	0.010	0.008

Note: Positive ΔAUROC favors the clinical model over the benchmark.

Supplemental Table S7. Transparent Ridge coefficients.

Feature	Coefficient (log OR)	Odds ratio
bin__History of Pre-op Heart Failure	0.977	2.655
num__Hemoglobin (g/dL)	-0.881	0.414
cat__Functional Capacity Assessment_≥4 MET	-0.755	0.470
num__Age	0.630	1.878
cat__Functional Capacity Assessment_<4 MET	0.482	1.620
cat__Sex_Female	-0.445	0.641
bin__missingindicator_Diabetes Mellitus (DM)	-0.409	0.664
num__missingindicator_Hemoglobin (g/dL)	0.389	1.475
bin__History of Myocardial Infarction (MI)	0.359	1.431
num__missingindicator_Creatinine (mg/dL)	-0.312	0.732
bin__Diabetes Mellitus (DM)	0.217	1.242
num__Creatinine (mg/dL)	0.164	1.178
cat__Functional Capacity Assessment_Unclear / not assessable	-0.143	0.866
bin__ESC_High_Risk	-0.080	0.923
cat__Sex_Male	0.029	1.029

Note: Odds ratios are on standardized or one-hot transformed features; they are not raw-unit odds ratios.

Supplemental Table S8. Algorithm implementation and hyperparameter specification.

Algorithm	Implementation	Non-default hyperparameters	Tuning
Ridge logistic regression	LogisticRegression (L2, liblinear)	C=0.72; class_weight=balanced; max_iter=5000	C tuned once in the development cohort: stratified 5-fold CV grid search, 8 log-spaced values 10^{-3}-10^{2}, log-loss criterion
Lasso logistic regression	LogisticRegression (L1, liblinear)	C=1.0; class_weight=balanced; max_iter=5000	None (fixed a priori)
Elastic-net logistic regression	SGDClassifier (logistic loss)	penalty=elasticnet; l1_ratio=0.5; alpha=10^{-3}; class_weight=balanced; max_iter=5000	None (fixed a priori)
Linear SVM	LinearSVC	class_weight=balanced; max_iter=10000	None (fixed a priori)
Random forest	RandomForestClassifier	n_estimators=400; min_samples_leaf=5;	None (fixed a priori)

		class_weight=balanced_subsample	
Gradient boosting	GradientBoostingClassifier	Library defaults	None (fixed a priori)
Histogram gradient boosting	HistGradientBoostingClassifier	Library defaults	None (fixed a priori)
AdaBoost	AdaBoostClassifier	Library defaults	None (fixed a priori)
Naive Bayes	GaussianNB	Library defaults	None (fixed a priori)
XGBoost	XGBClassifier	n_estimators=300; max_depth=3; learning_rate=0.05; subsample=0.8; colsample_bytree=0.8; eval_metric=logloss	None (fixed a priori)

Note: All models: probability outputs calibrated with Platt (sigmoid) calibration fitted by internal 5-fold splitting within the development cohort; class imbalance handled with balanced class weights where supported; fixed random seed throughout. The external validation cohort was used for no tuning, selection, calibration, or threshold decision. Implementations: scikit-learn; XGBoost via the xgboost library.

Supplemental Figures

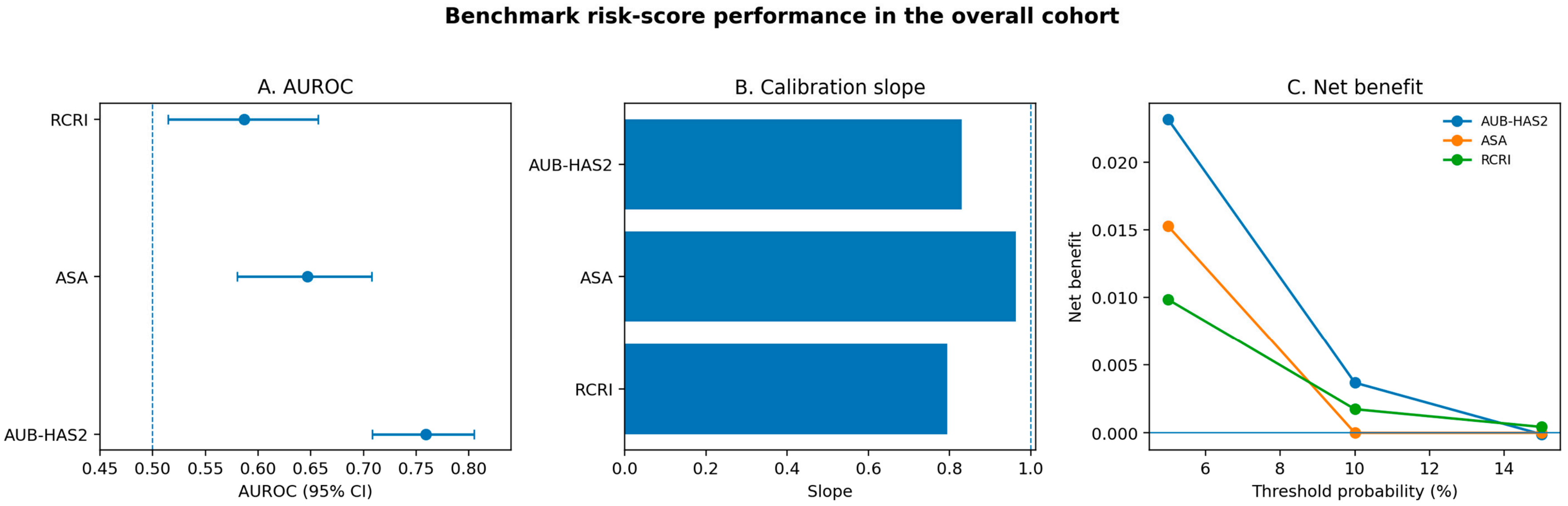

Supplemental Figure S1. Benchmark risk-score performance in the overall cohort.

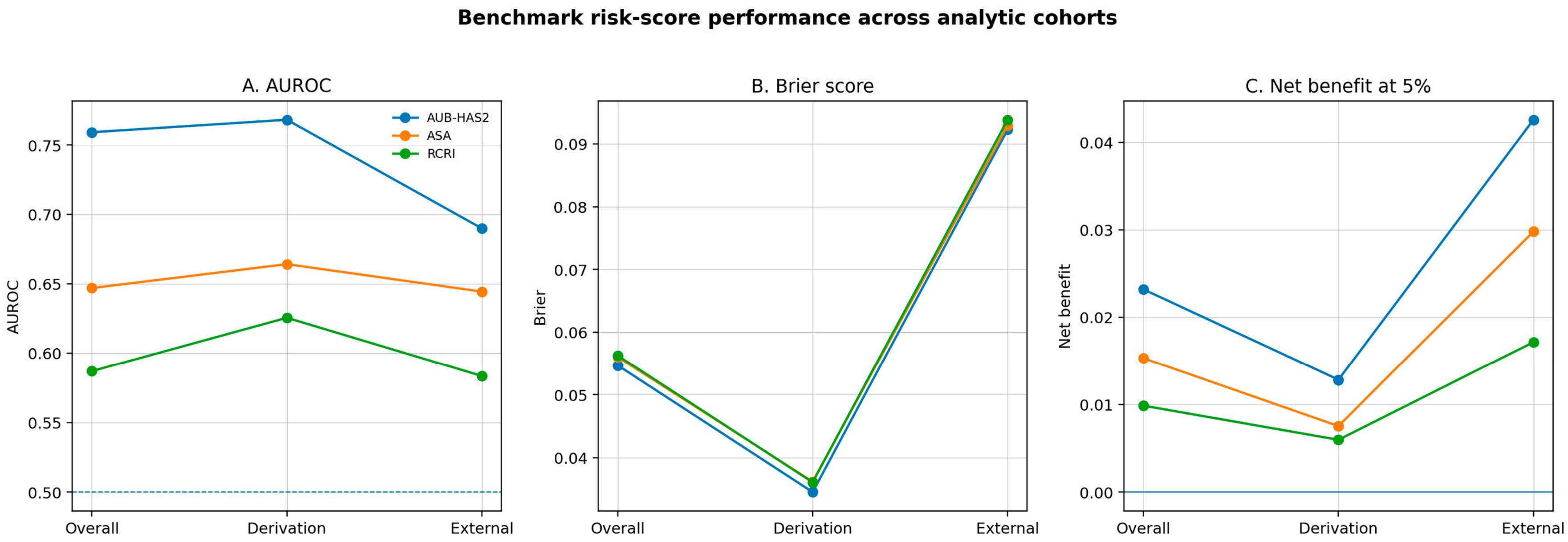

Supplemental Figure S2. Benchmark risk-score performance across the overall, derivation, and external validation cohorts.

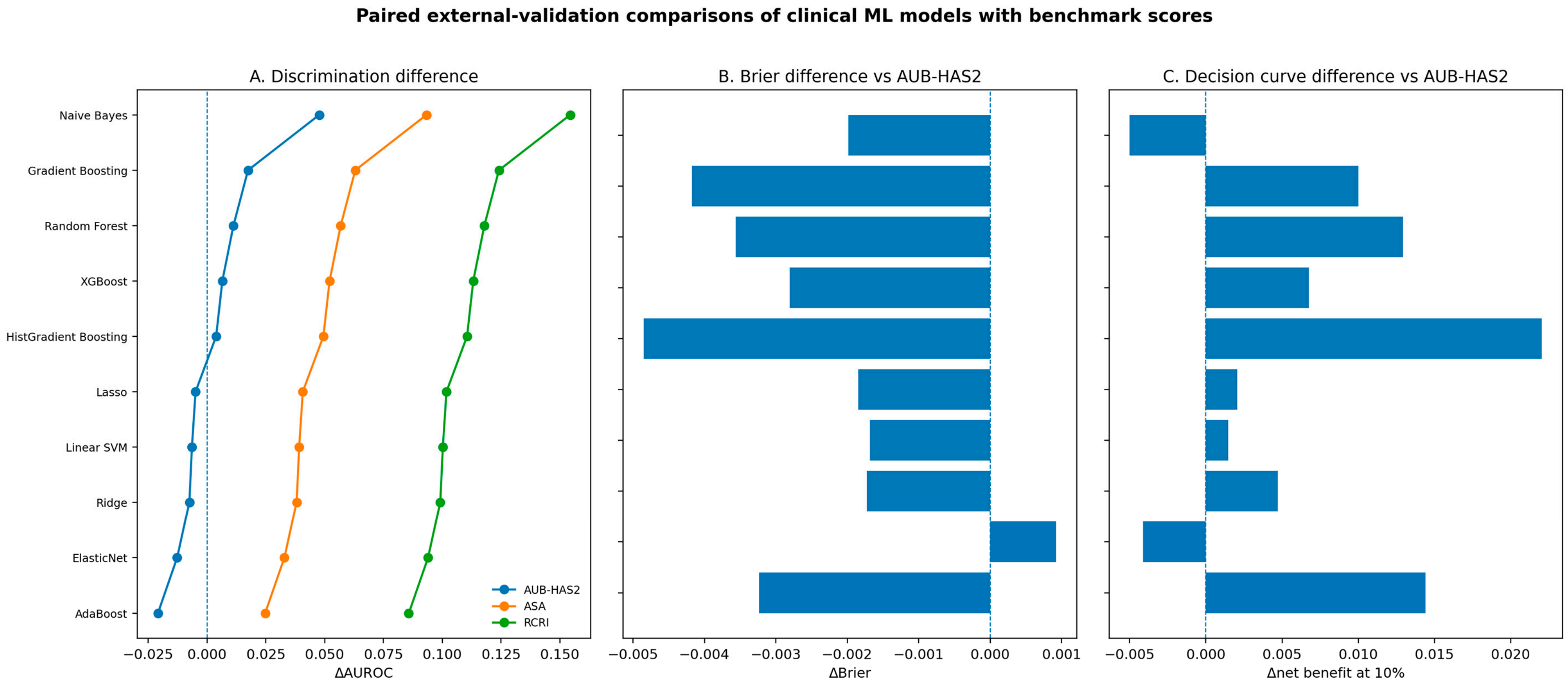

Supplemental Figure S3. Paired external-validation comparisons of clinical ML models with benchmark scores.

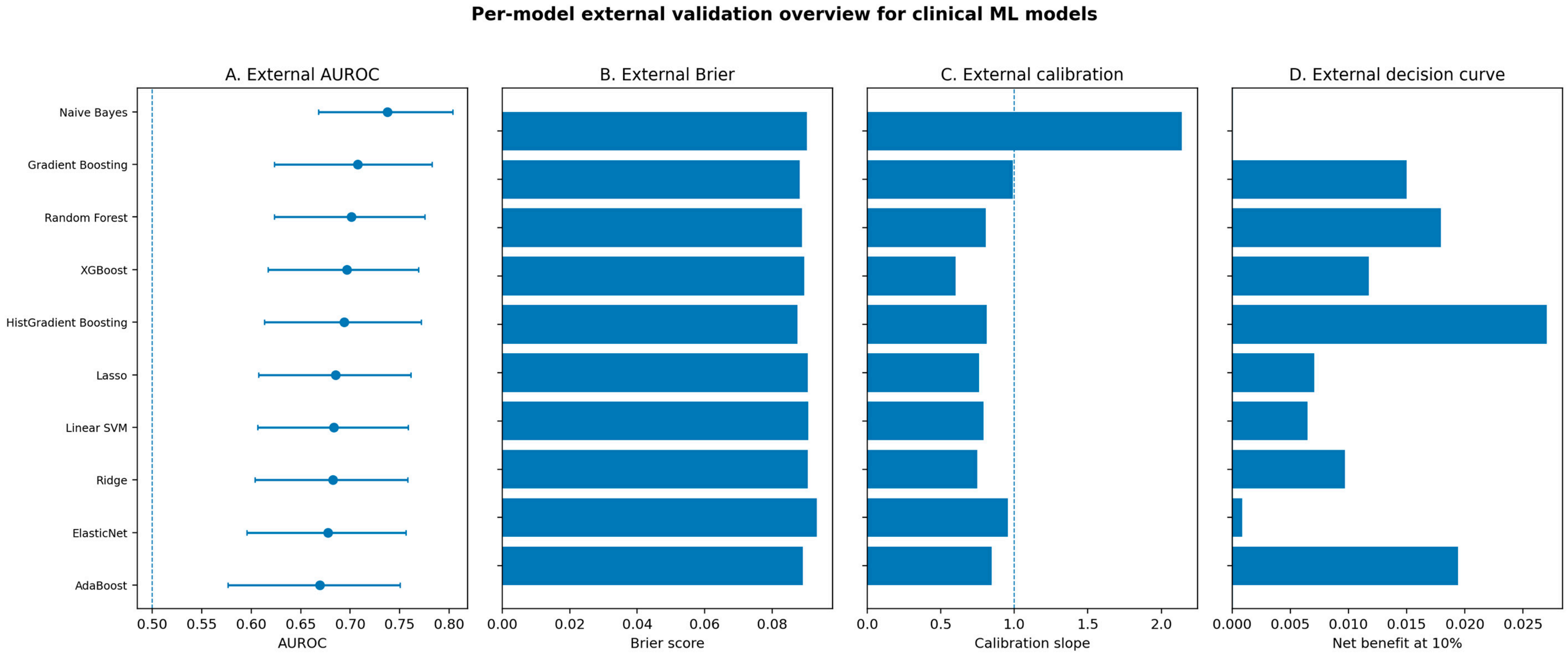

Supplemental Figure S4. Per-model external validation overview for clinical ML models.
